# The survey and reference assisted assembly of the *Octopus vulgaris* genome

**DOI:** 10.1038/s41597-019-0017-6

**Published:** 2019-04-01

**Authors:** Ilaria Zarrella, Koen Herten, Gregory E. Maes, Shuaishuai Tai, Ming Yang, Eve Seuntjens, Elena A. Ritschard, Michael Zach, Ruth Styfhals, Remo Sanges, Oleg Simakov, Giovanna Ponte, Graziano Fiorito

**Affiliations:** 1 Association for Cephalopod Research ‘CephRes’, Napoli, Italy; 2 Genomics Core, KU Leuven, Leuven, Belgium; 3 Laboratory for Cytogenetics and Genome Research, Center for Human Genetics, KU Leuven, Leuven, Belgium; 4 Laboratory of Biodiversity and Evolutionary Genomics, KU Leuven, Leuven, Belgium; 5 Centre for Sustainable Tropical Fisheries and Aquaculture, Comparative Genomics Centre, College of Science and Engineering, James Cook University, Townsville, 4811 QLD Australia; 6 BGI Genomics, BGI-Shenzhen, Shenzhen, 518083 China; 7 Laboratory of Developmental Neurobiology, Department of Biology, KU Leuven, Leuven, Belgium; 8 Department of Molecular Evolution and Development, University of Vienna, Vienna, Austria; 9 Department of Biology and Evolution of Marine Organisms, Stazione Zoologica Anton Dohrn Napoli, Napoli, Italy

**Keywords:** Genome evolution, Genome, DNA sequencing, Marine biology

## Abstract

The common octopus, *Octopus vulgaris*, is an active marine predator known for the richness and plasticity of its behavioral repertoire, and remarkable learning and memory capabilities. Octopus and other coleoid cephalopods, cuttlefish and squid, possess the largest nervous system among invertebrates, both for cell counts and body to brain size. *O*. *vulgaris* has been at the center of a long-tradition of research into diverse aspects of its biology. To leverage research in this iconic species, we generated 270 Gb of genomic sequencing data, complementing those available for the only other sequenced congeneric octopus, *Octopus bimaculoides*. We show that both genomes are similar in size, but display different levels of heterozygosity and repeats. Our data give a first quantitative glimpse into the rate of coding and non-coding regions and support the view that hundreds of novel genes may have arisen independently despite the close phylogenetic distance. We furthermore describe a reference-guided assembly and an open genomic resource (CephRes-gdatabase), opening new avenues in the study of genomic novelties in cephalopods and their biology.

## Abstract (summary)

**Table:** 

Design Type(s)	species comparison design • sequence analysis objective • sequence assembly objective
Measurement Type(s)	whole genome sequencing assay
Technology Type(s)	DNA sequencing
Factor Type(s)	
Sample Characteristic(s)	Octopus vulgaris • testis • ocean biome

Machine-accessible metadata file describing the reported data (ISA-Tab format)

## Background & Summary

*Octopus vulgaris* is a benthic, neritic species belonging to the class Cephalopoda. It occurs from the coastline to the outer edge of the continental shelf, inhabiting various marine habitats at depths spanning from 0 to 200 m. *O*. *vulgaris* is one of the most widely distributed species belonging to the genus, and is an important commercially harvested resource for human consumption. It is found worldwide in temperate and tropical waters^1–3^. Throughout its distribution range, the animal undertakes limited seasonal migrations: mostly found in deep waters in winter and shallow waters in summer.

*O. vulgaris* is perhaps the most famous and best studied of all octopus species, largely due to the initiative of Professor John Z. Young^4,5^. Since the late 1940 s, Young carried out at the Stazione Zoologica Anton Dohrn of Napoli (Italy) a systematic analysis of the neural structures underlying behavioural plasticity in this animal^6,7^. Based on this contribution, the anatomy of *O. vulgaris* nervous system^8^ and its physiology and life history^4,9–11^ have been well characterized. It is the phenomenological proximity of behavioral traits and phylogenetic distance in respect to higher vertebrates that guaranteed the short, but wide success of cephalopods^5,10^. *O. vulgaris* in particular became a “model of the brain”^12,13^, and more recently a case for studying the evolution of cognition in invertebrates^7,14–19^. Researchers still use *O. vulgaris* as an organism to study behavioural and neural plasticity including learning and memory recall^5,12,20^, regeneration^21–24^ and sophisticated cognition^7,14–17,25^.

Currently available genomic resources for molluscs are scarce, considering the species abundance and the commercial value of the phylum Mollusca. Publicly available molluscan genomes include a dozen representatives from bivalves, gastropods^26–42^ and to-date only three cephalopods, namely the California two-spot octopus *Octopus bimaculoides*^43^ and, more recently, for *Callistoctopus minor*^44^ and *Euprymna scolopes*^45^.

Although the first step towards cephalopod genetics was made over 30 years ago^46^, cephalopod research is only slowly entering the genomics era^10,47^. Obtaining high quality cephalopod genomes has been impeded due to their large size (e.g., *O. bimaculoides*: 2.7 Gb; Gregory, 2018 - Animal Genome Size Database, http://www.genomesize.com), heterozygosity and high abundance of repeat regions^43,47,48^. However, several collaborative genome projects are currently underway for a variety of cephalopod species such as the nautilus, *Sepia officinalis, Idiosepius paradoxus* and *Doryteuthis pealeii*.

Cephalopods arose more than 500 Mya and diverged into over 800 current living species with highly diversified life styles and body plans^48^. Translocations, duplications, exon shuffling and gene conversions occurred within the cephalopod genome during evolution, which might explain the development of different morphological novelties, such as the prehensile arms, the unique jet propulsion system, the ink sac and sophisticated sensory and neural systems^49^. The analysis of *O. bimaculoides* genome revealed an extensive expansion of particular gene families, including protocadherins and the C2H2 superfamily of zinc-finger transcription factors^43^, as well as novel octopus-specific genes expressed in specialized structures such as suckers, skin and brain (for review see also Shigeno *et al*.^18^). These genome-level novelties are accompanied by other sophisticated innovations such as extensive RNA editing, particularly in the nervous system cells^50–52^. Furthermore, partial genome sequencing of several cephalopods showed that repeat elements, in particular transposable elements, are abundant^53,54^. Indeed, the genome of *O. bimaculoides* revealed that over 45% of the genome is comprised of repetitive elements^43^.

The study of cephalopod biological innovations^10,18,43,55^ is driven by the unique scientific value of these animals for evolutionary genomics, neuroscience and cognition^7,10,18,25,43,55–58^ which continues the heritage of the discovery of the action potential in the squid giant axon, a seminal contribution to neuroscience^59^. Furthermore, the phylogenetic relationships within the cephalopods have not yet been fully elucidated and biological research would benefit from more cephalopod genomes^60,61^.

In line with those previous and current efforts, and to promote data sharing among cephalopod researchers^10,47^, we present the sequence and draft assembly of the common octopus, *Octopus vulgaris*, genome. It is noteworthy to report that the two species (*i.e*., *O. vulgaris* and *O. bimaculoides*), although both belonging to the same genus, go through a substantially different life cycle since the paralarval stage is absent in *O. bimaculoides*^62^. Therefore, the two species represent different biological and physiological adaptations among closely related species. The genomic sequencing of both octopus species and our online platform to browse these data will allow for future comparative genomics studies, revealing key genomic innovations and facilitating the discovery of the molecular basis of intricate processes such as learning, regeneration and the evolution of complex brains.

## Methods

### Genomic DNA preparation

An adult male belonging to the species *O. vulgaris* Cuvier, 1797 (450 g body weight) was caught by fishermen from the Bay of Naples in 2011^1,2^ and immediately humanely-killed^63,64^. Given the high rate of heterozygosity in marine organisms^65,66^, tissue from a single individual was used to extract the genomic DNA (to avoid contamination, spermatophores were used). Spermatophores in octopus are stored within the Needham’s sac, structure that was dissected following Chapko and coworkers^67^. Tissue (124 mg) was used to extract the genomic DNA following the recommended phenol-chloroform extraction protocol by the Beijing Genomics Institute (BGI)-Shenzhen. Briefly, tissue lysis occurred overnight at 56 °C after adding 3.0 ml of lysis buffer containing proteinase K (300 μg; Sigma-Aldrich, Saint Louis, Missouri, United States) and RNase A (100 μg; Sigma-Aldrich, Saint Louis, Missouri, United States). DNA was then extracted with phenol (2X), phenol:chloroform, chloroform and was subsequently precipitated. Genomic DNA was dissolved in TE buffer to reach a final concentration of 1 μg/μl.

### Genome sequencing and quality control

A total of four genomic DNA libraries (with different insert sizes: 170, 250, 500 and 800 bp) were constructed following the Illumina library preparation protocols. Briefly, to construct the paired-end libraries DNA was fragmented by Adaptive Focused Acoustics technology (Covaris) and tested via gel-electrophotometry, the fragmented DNA combined with End Repair Mix (20 °C for 30 min). After purification, DNA ends were blunted and an A base was added to the 3′ ends. DNA adaptors with a single T-base 3′-end overhang were ligated to the above products. Ligation products were purified on 2% agarose gels to recover the target fragments and were purified from the gels (Qiagen Gel Extraction kit, 28704). Several rounds of PCR amplification with PCR Primer Cocktail and PCR Master Mix were performed to enrich the Adapter-ligated DNA fragments. Then the PCR products selected by running another 2% agarose gel to recover the target fragments and the gel purified (QIAquick Gel Extraction kit, QUIAGEN). The final library was quantified by assessing the average molecule length (Agilent 2100 Bioanalyzer), and by Real-Time qRT-PCR. A total of 277 Gb of raw data were generated by Illumina Hiseq 2000 at BGI.

All libraries were sequenced in a paired-end mode with read lengths of 100 bp or 150 bp. Reads were filtered and trimmed (100 bp to 95 bp, 150 bp to 145 bp) using SOAPnuke software (https://github.com/BGI-flexlab/SOAPnuke)^68^ which yielded 250 Gb of data. Low-quality reads, reads with adaptor sequences and duplicated reads were filtered, and if the quality of bases at the head or tail of the reads was low, we directly trimmed them from 100 bp to 95 bp (PE100) or form 150 bp to 145 bp (PE150). The remaining high-quality data were used in the further analysis. *SGA PreQC v0.10.14*^69^ modules were run per library and on the combined libraries to estimate various genome parameters (Table 1 and Table 2).

**Table 1:** Main statistics from *O.*

Library ID	Insert Size(bp)	Read Length (bp)	Data (Gb)	Sequence Depth (X)
SZAXPI006102-158	170	100	82.15	29.34
SZAXPI006612-13	250	150	52.25	18.66
SZAXPI005989-166	500	100	62.05	22.16
SZAXPI005988-169	800	100	53.59	19.14
Total	—	—	250.04	89.30

*vulgaris* sequencing data.

**Table 2:** k-mer = 17 raw read statistics for *Octopus vulgaris* genome data.

K-mer_num	Peak_depth	Genome Size	Used Bases	Used Reads
212,679,899,304	76	2,798,419,727	249,873,643,000	2,324,608,981

### Draft genome assembly

We applied Assembly By Short Sequencing 2.0.2 (ABySS^70,71^) for both k-mer sizes that were suggested by *SGA PreQC*. The quality of assemblies (ABySS kmer41 and ABySS kmer81) was evaluated by QUAST 4. 3^72^. A summary of various statistics is shown in Table 3. Based on the *QUAST* analysis the optimal kmer size for the ABySS assembly was estimated to be 81. Since a higher heterozygosity rate of the genome was predicted based on these initial results, the *Redundans* 0.13 c^73^ tool was used to reduce the number of ABySS contigs from the initial assemblies. *Redundans* reduces contigs by removing highly similar contigs. These highly similar contigs are originally the different alleles of the same genomic position, but are too different for the De Brujin graph method to be assembled into the same contig (too much variation inside one kmer). *Redundans* collapses and scaffolds these reduced contigs into single genomic locations. *Redundans* reduced the number of scaffolds of the draft genome over seven (7) times, while improving assembly statistics (see Table 3).

**Table 3:** Assembly statistics for *Octopus vulgaris.*

	# scaffolds	genome size	N50/L50	N75/L75	Ns/100 kbp	Complete BUSCOs	Fragmented BUSCOs
ABySS k41 scaffolds	26,350,077	3,30 Gb	1,488 bp 199,442	767 bp 503,977	979.41	112	50
ABySS k81 scaffolds	8,918,381	3.31 Gb	2,627 bp 195,104	980 bp 496,991	706.92	275	286
Redundans k81	1,157,969	2.10 Gb	3,958 bp 149,577	2,126 bp 330,514	3,961.18	390	319
Chromosomer k81	77,683	1.78 Gb	263,097 bp 1,607	56,379 bp 5,018	19,504.19	505	88
*O. bimaculoides*	151,674	2.34 Gb	485,615 bp 1,300	215,581 bp 3,077	15,346.35	773	28

Statistics were generated with QUAST and a default threshold of 500 bp. See text for details.

### Reference Assisted Scaffolding

Given the availability of a relatively good reference genome of a related species (*O. bimaculoides*)^43^, a reference assisted scaffolding tool was used to optimize the genome. The reduced scaffolds were aligned to the *O. bimaculoides* genome using blastn^74^ of the blast+ toolkit 2.8.0-alpha. These alignments were used by *chromosomer* 0.1.3 (https://github.com/gtamazian/Chromosomer) to scaffold the reduced scaffolds according to the given genome.

### Assessment of draft genomes

An assessment of the draft genomes (ABySS, Redundans and chromosomer) was performed by looking for the highly conserved genes using BUSCO 3.0. 2^75^. The Metazoa odb9 database was used, supplying 978 orthologs. The number of complete orthologs increased with each improvement of the assembly (Table 3), confirming the gain in assembly quality of the final chromosomer version. The final genome build has over 50% complete BUSCOs, and 10% fragmented BUSCOs (orthologs found, but scattered over multiple scaffolds).

## Data Records

**Fig. 1:**
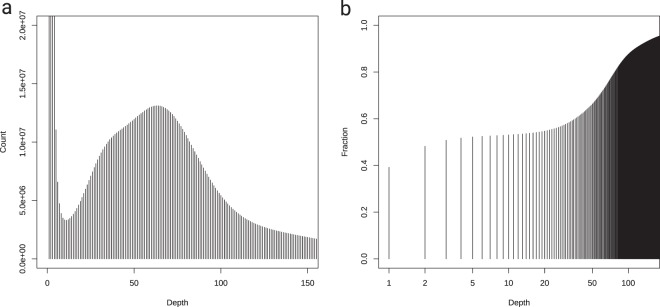
Sequencing depth and genome repetitiveness estimation from 17mer counts in the raw read data. (**a**) 17mer depth analysis using raw data showing elevated levels of heterozygosity. (**b**) Cumulative proportion of 17mers as a function of their depth showing that at least half of the genome occurs at depth 10 or more.

The draft genome(s) of *O. vulgaris* as shown in Table 3 has been made publicly available on the genome browser and data repository of the Association for Cephalopod Research that initiated this work (http://www.cephalopodresearch.org/ceph_gdatab/) in collaboration with the Department of Molecular Evolution and Development, University of Vienna. This web resource is based on the browser originally designed by University of California, Santa Cruz (UCSC)^76^ and will be maintained and curated to keep track of all present and upcoming octopus genomes. It includes comparative genomics tracks such as read mapping and whole genome alignment between the two octopus species. Raw reads have also been deposited to the NCBI SRA^77^. The reference-guided assembly has been deposited at GenBank^78^ and its original version is also provided in the associated FigShare record (chromosomer.fa) together with its annotation (gene_models.chromosomer.gff), and other assemblies listed in Table 3 (*Octopus vulgaris* genome assemblies^79^. Table 2 and Table 3 summarize statistics about *O. vulgaris* genome as deduced from our current sequencing data and Fig. 1 shows the kmer (17mer) distribution determining the overall sequencing depth (Table 1 and 2).

## Technical Validation

### Quality control

The quantity and integrity of the genomic DNA was analysed via agarose gel electrophoresis and with a NanoDrop spectrophotometer (Thermo Fisher Scientific; concentration of 1 μg/μl, A_260_/A_*280*_ = 1.84 and A_230_/A_260_ = 2.2). DNA integrity was analysed with Agilent Bioanalyzer 2100.

### Quality control DNA library

To assess the quality of Illumina reads FastQC (www.bioinformatics.babraham.ac.uk/projects/fastqc) was performed on all raw data. *Trimmomatic v0.36*^80^ was was not able to identify any significant adaptor sequence contamination within the raw data. The data were mapped to the PhiX control library (Illumina, Inc) using Bowtie2 v2.3.4^81^ and no matches were found.

### Sequencing depth assessment

We used jellyfish 2.2. 10^82^ on the raw read data using kmer size of 17 bp. This resulted in a depth of sequencing histogram (Fig. 1) showing sequencing depth peak of around 76x. Using the kmer depth curve and the cumulative read depth (Fig. 1), repetitiveness, and heterozygosity was conducted independent of the genome assemblies (see Tables 2 and 3). The genome was estimated to be around 2.4 Gb in length with a relatively high heterozygosity rate (>1.1%) and large repetitiveness (>50%).

## Genome properties and future steps

**Fig. 2:**
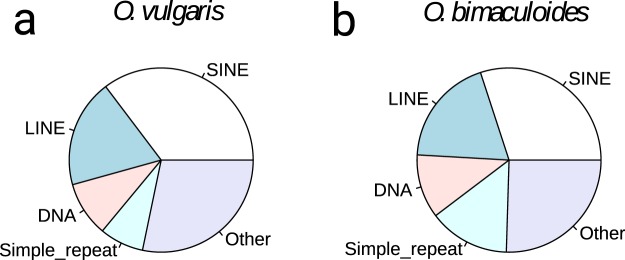
Proportions of the most abundant repetitive element classes in *Octopus vulgaris* compared to *Octopus bimaculoides* based on the *ab initio* reconstruction of repetitive elements using the DNAPipeTE pipeline. (**a**) Repeat propotions in the *Octopus vulgaris* genome. (**b**) Repeat propotions in the *Octopus bimaculoides* genome. In both genomes, SINE elements are the most abundant repeat classes. While the total number of repeats is similar in both genomes, differences in the proportions can be attributed to individual expansions of repeat elements that occurred independently in both lineages.

To gain information on the genetic distance between the two closely related species *O. vulgaris* and *O. bimaculoides*, we mapped all the available raw sequence data from *O. vulgaris* against the genome of *O. bimaculoides*^83^ and found that 74–84% of the data aligned, but that a high percentage (20–50%) was able to align multiple times. The significant proportion of multiple mapping reads suggests that, similar to the *O. bimaculoides* genome, *O. vulgaris* genome has a large number (at least 50%) of repetitive elements, confirmed by the cumulative read depth analysis (Fig. 1). *Ab initio* repeat analysis using dnaPipeTE^84^ revealed similar classes of octopus specific short interspersed nuclear elements (SINE) to be over-represented (Fig. 2), yet the proportions were strikingly different, despite the close phylogenetic distance. This indicates high activity of repetitive elements in the common octopus genome.

**Fig. 3:**
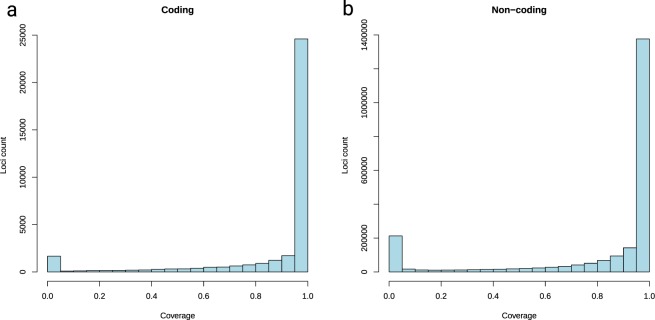
Comparison of coding and non-coding region conservation between *Octopus bimaculoides* and *Octopus vulgaris*. (**a**) Alignment coverage in the coding genomic regions. (**b**) Alignment coverage in the non-coding, non-repetitive genomic regions. Coverage shows the proportion of nucleotides that are covered in *O. bimaculoides* assembly with *O. vulgaris* read mapping in both coding and non-coding non-repetitive regions of at least 100 bp. The main peak at 1 (100% coverage) indicates the presence of a complete region in *O. vulgaris* genome at very low sequence divergence, whereas the secondary peak at 0 indicates regions of *O. bimaculoides* genome that are not matching in *O. vulgaris* read data (see text for analysis).

Profiling *O. bimaculoides* regions with read coverage from *O. vulgaris*, we found that 23,509 *O. bimaculoides* genes were covered at 90% or more of their coding sequence length by *O. vulgaris* reads (Fig. 3). Approximately 50% of those genes had a Pfam annotation, including gene families previously reported to have undergone major expansions in the *O. bimaculoides* genome, such as zinc fingers and protocadherins. This is in strong contrast to only 1,570 *O. bimaculoides* genes with no *O. vulgaris* read coverage, with just 14% of those having a Pfam annotation. Those candidates represent very recent novel or highly diverged genes and their number indicates a relatively high rate of novel gene formation in octopus genomes. To investigate non-coding evolution among cephalopods, we furthermore compared the mapping rates to non-repetitive non-coding regions of 100 bp and longer. Again, we found the majority of those loci are covered at 90% length or higher. However, the relative proportion of *O. bimaculoides* regions not covered by any reads was higher than for the genes, indicating a higher turnover rate for the non-coding, potentially regulatory, sequences (Fig. 3).

To evaluate the completeness of our assemblies, raw reads were mapped using Bowtie2 v2.3.4 against both ABySS kmer81 and kmer41 assemblies. For ABySS kmer 41, at least 99.94% of all the reads were mapped while the percentage of uniquely mapped reads was only around 33–50%. For the ABySS kmer81 assembly, percentages were at least 98% and between 31 and 57%, respectively.

**Fig. 4:**
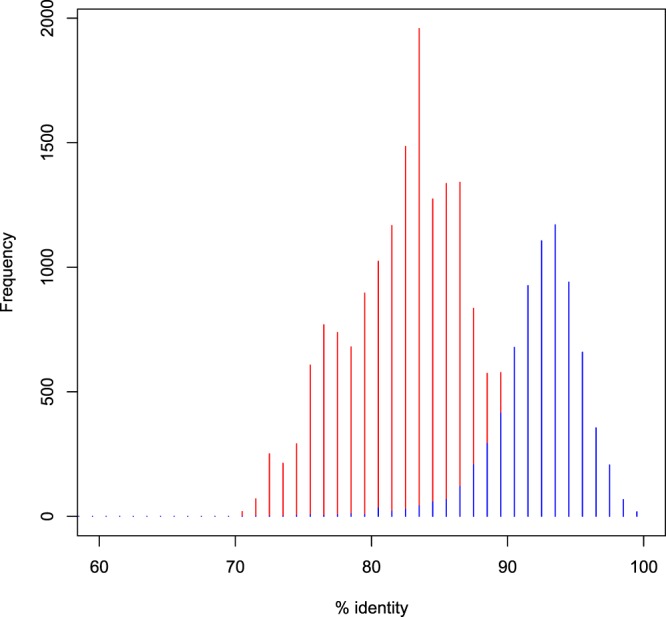
Comparison of whole genome alignments using MEGABLAST among the available octopod genomes. Only the longest scoring alignment between any given pair of two scaffolds or contigs was considered. Red: percentage nucleotide identity between *Callistoctopus minor* to *Octopus bimaculoides*. Blue: percentage nucleotide identity between *Octopus vulgaris* to *O. bimaculoides.*

We used our assemblies to estimate whole-genome divergences between the available octopod genomes. Mapping of the scaffolds of 10 kb and longer against the *O. bimaculoides* genome using MEGABLAST resulted in the overall sequence similarity of 92.4% in the aligned regions of 1 kb and above (Fig. 4). This divergence of around 8% between the two species is higher than the estimated heterozygosity rate of 1.1% in *O. vulgaris* and lower than the divergence between *O. bimaculoides*^83^ and the recently released data of *C. minor* (82.4% similarity) (Fig. 4, and ref.^85^) from a different genus, providing for the first whole-genome divergence estimates within this clade.

Our assemblies confirm that abundant repeat regions make it difficult to improve the genome based on the currently available sequence data. Future steps will include long read sequencing technology such as proximity-ligation based assemblies (*e.g*., Dovetail, PhaseGenomics) or longer read technologies (*e.g*., PacBio) to optimize the current assemblies.

## ISA-Tab metadata file

Download metadata file
